# Re-irradiation of Pediatric Medulloblastoma: A Case Report and Systematic Review

**DOI:** 10.7759/cureus.31585

**Published:** 2022-11-16

**Authors:** Georgios Giakoumettis, Artemis Mantzavinou, Georgios Moschos, Dimitrios Giakoumettis, Antonio Capizzello

**Affiliations:** 1 Medical Physics, Aristotle University of Thessaloniki, Thessaloniki, GRC; 2 Medicine, Barts and The London School of Medicine and Dentistry, London, GBR; 3 Department of Radiation Oncology, AHEPA University Hospital, Thessaloniki, GRC; 4 Neurosurgery, Queen's Hospital, Romford, London, GBR

**Keywords:** posterior fossa mass, pediatric brain tumour, reirradiation, radiotherapy, medulloblastoma

## Abstract

Despite the optimal treatment given to children with medulloblastoma, many relapses are seen after combining treatments. Re-irradiation is part of salvage therapy for children who relapse and might provide long-term disease control. Nevertheless, it is challenging because there is a concern about exceeding radiation tolerances and late treatment toxicities. Re-irradiation is an option for many brain tumors, including medulloblastoma in children. This study presents a case of recurrent medulloblastoma treated with re-irradiation. A systematic review of the literature provided up-to-date data on the re-irradiation of medulloblastoma in children. This study aims to contribute to the scarce literature on the treatment strategy, which may help improve patients' outcomes.

## Introduction

Medulloblastoma (MB) is the most common malignant pediatric tumor of the Central Nervous System (CNS), accounting for 20% of childhood brain cancers. It is a malignant primitive embryonal tumor that typically arises in the cerebellum [1]. Standard treatment for patients over three years old involves a surgical operation with maximal safe resection followed by adjuvant radiotherapy and/or chemotherapy. Radiotherapy includes craniospinal irradiation (CSI) and "boost" radiotherapy to the posterior fossa or tumor bed, followed by platinum-based chemotherapy. This treatment has resulted in five-year overall survival of 85% and 70% for average-risk and high-risk diseases, respectively [2].

Despite this therapy, at least 20-30% of patients will have a relapse. Recurrence represents the commonest cause of death in patients with medulloblastoma. The expected two-year overall survival (OS) after disease progression is less than 25%. Management of these patients has been challenging as there is no standard approach to salvage therapy, including repeat surgery, systemic therapy, high-dose chemotherapy with stem cell rescue, and repeat radiotherapy (RT) [3].

The majority of patients with recurrent medulloblastoma are usually treated with craniospinal irradiation [4]. The re-irradiation of these patients can prove challenging due to a concern of exceeding radiation tolerances and late treatment toxicities. Normal brain tissue can tolerate a total dose of 100 Gy; therefore, re-irradiating these patients risks exceeding this limit and consequently leading to brain tissue necrosis [5]. Re-irradiation is an option for other types of brain tumors, but to the author’s knowledge, there is a lack of data in the literature about the re-irradiation of recurrent medulloblastoma in children [6].

## Case presentation

**Figure 1:**
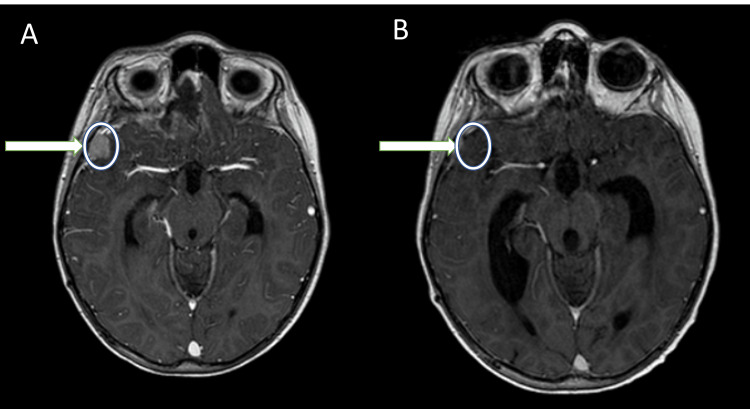
Axial Images of MRI T1WI sequence with contrast at the same level. A: Recurrence of medulloblastoma, the new site at the right temporal pole. B: Three months after focused re-irradiation of the tumour.

A four-year-old boy presented with headache and neck pain in the neurology clinic. He was found to have cerebellar ataxia physical examination. Cranial Magnetic Resonance Imaging (MRI) showed a contrast-enhancing lesion in the fourth ventricle causing obstructive hydrocephalus, for which the patient underwent an external ventricular drainage (EVD) procedure. Subsequently, the patient underwent a surgical operation where a subtotal removal of the tumor ( > 1.5 cm^2^ residual disease) was achieved. Histology examination was positive for classic-type medulloblastoma, but no molecular profiling was performed. Cerebrospinal fluid cytology examination was negative, and a spine MRI with contrast reported several hyperintense areas in the T2-weighted sequence with radiological features suspicion of neoplastic disease. After surgery, the patient received chemotherapy and craniospinal radiation therapy. Radiotherapy treatment was performed using proton therapy. The total dose to the brain and spinal axis was 35.20 Gy plus a sequential boost to the metastatic lesions of the spine for a total dose of 46 Gy and a sequential boost to the posterior fossa for a total dose of 55 Gy. The patient did not show any recurrence in his follow-up for three years. After three years, his brain MRI showed the presence of a new gadolinium-enhanced mass in the right sub-frontal region. No signs of tumor recurrence in the spinal canal or the posterior cranial fossa were noticed. Findings on physical examination included postural and gait instability, left-sided hemiparesis, dysarthria, and neuropsychological symptoms. Gross total resection of the right frontal tumor was achieved with right frontal craniotomy and confirmed with a postoperative CT of the brain with contrast at 24 hours. Immunohistochemistry of the excised tumor revealed a recurrent medulloblastoma. A follow-up MRI with gadolinium contrast of the whole neuroaxis (brain and spine) at six weeks showed a new contrast-enhanced mass in the right temporal pole (Figure 1A). Subsequently, the case was discussed in the neuro-oncology multidisciplinary team (MDT) meeting and the outcome was to follow oncological management and not a surgical operation. Accordingly, the temporal pole mass and surgical bed were treated with synchronous radiotherapy and chemotherapy (temozolomide). Radiotherapy treatment was performed using Volumetric modulated arc therapy (VMAT) with a simultaneous integrated boost (SIB) technique. A total radiation dose of 50.6 Gy in 22 fractions (2.3 Gy per fraction) was delivered at the new gadolinium-enhanced mass in the right temporal pole, and simultaneously, a total dose of 39.6 Gy in 22 fractions (1.8 Gy per fraction) targeted the surgical bed. A thermoplastic mask for head immobilization was used for CT simulation. Target volume delineation was performed through MRI-image fusion, applying to the gross tumor volume (GTV) a 3-mm margin overlapping the brainstem, which was given a higher priority during the planning phase. The planning target volumes (PTV) were 15.522 cm^3^ for the new mass and 233.170 cm^3^ for the surgical bed. According to the treatment plan, all organs at risk were protected; the maximum dose calculated for the brainstem was 18.428 Gy and for the brain tissue was 24.975 Gy. Daily kilovoltage (kV) CT scan imaging before each fraction was applied. Informed consent was obtained before the start of the treatment. The patient tolerated the treatment without severe (grade ≥3) acute symptoms, according to the Radiation Therapy Oncology Group (RTOG) system. Acute toxicities reported were fatigue (n=2), alopecia (n=2), and nausea (n=1). Improvement was noted in hemiparesis and instability. After three months, a brain MRI reported no evidence of disease (Figure 1B), and the patient was well and in good general condition at his three-year follow-up.

Methods

Two independent researchers performed a systematic review of the literature in the PubMed database according to Prisma guidelines [7]. We used an advanced search with boolean logic under the search terms 'irradiation' or 'radiotherapy' or 're-irradiation' and 'medulloblastoma'. We applied filters in our search: a ten-year period (2011 to 2022) and the language of the articles English, French, and German. We included in our search children 0-18 years, metastatic brain medulloblastoma, re-irradiation, and recurrent medulloblastoma, and we excluded reviews, adults, and any article that does not meet the inclusion criteria & duplicates.

Results

**Figure 2:**
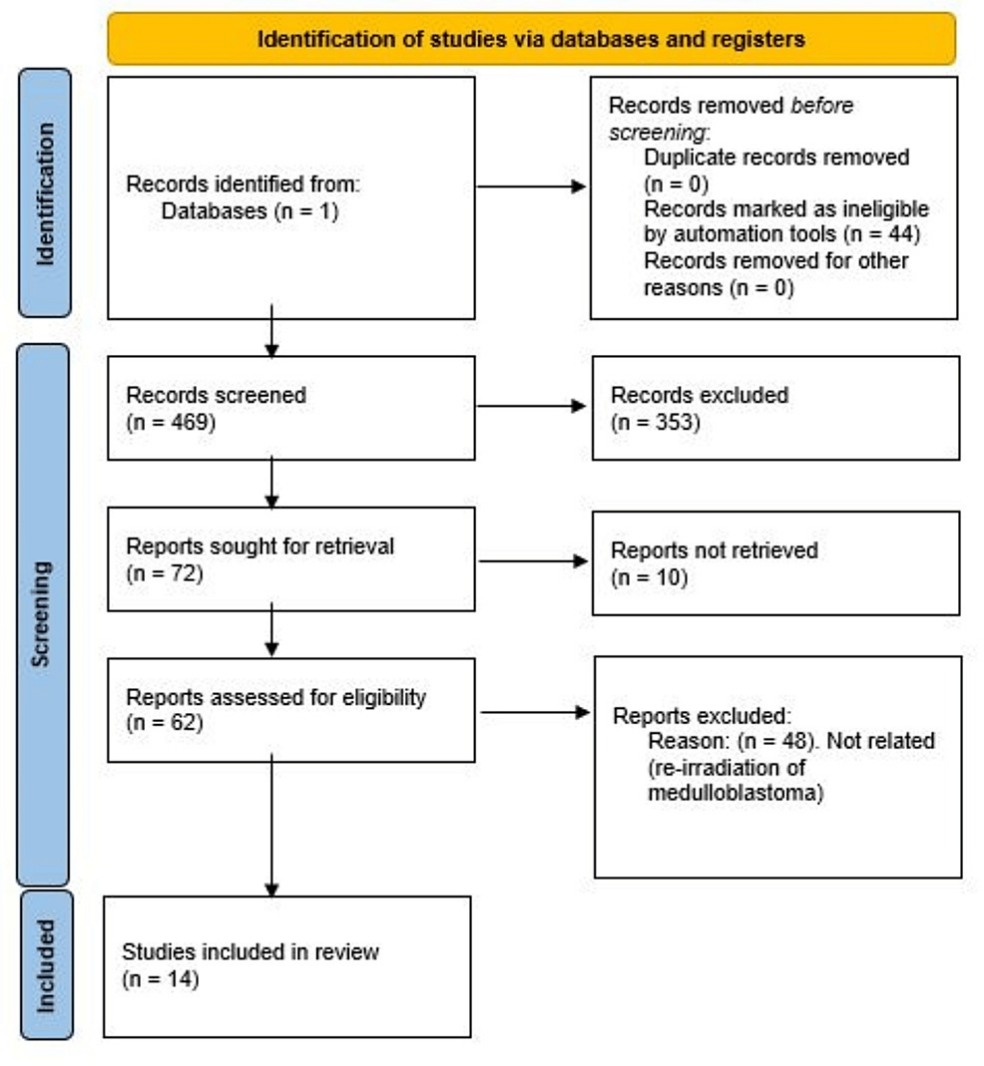
Preferred Reporting Items for Systematic Reviews and Meta-Analyses (PRISMA) Flow Chart

Our search yielded 469 articles, and titles and abstracts were screened for eligibility. Of the total number of 469 articles, a number of 353 met our exclusion criteria, and thus, they were excluded from our analysis. A total number of 14 studies were included in our analysis, and the full text was retrieved and assessed. The search outcomes and screening procedure are in the PRISMA flow diagram in Figure 2 [7]. Table 1 presents a complete list of the articles that were retrieved and included in our study.

**Table 1:** A complete list of the articles with the outcomes that were retrieved and included in the study.

No.	AUTHORS	TITLE	YEAR	PMID	OUTCOME
1	Derek S. Tsang et al. [8]	Re-irradiation for children with recurrent medulloblastoma in Toronto, Canada: a 20-year experience.	2019	31468270	Re-irradiation was adequate and well-tolerated, offering disease control for children with focal recurrent cerebral medulloblastoma. The combination of re-irradiation, systemic rescue therapy and stem cell rescue can provide long-term survival. Survival may depend on different molecular subgroups of medulloblastoma.
2	Tejpal Gupta et al. [9]	Outcomes of salvage re-irradiation in recurrent medulloblastoma correlate with age at initial diagnosis, primary risk-stratification, and molecular subgrouping.	2019	31236820	Young patients with average-risk disease at initial diagnosis and molecular subgroup four are best suited for re-irradiation, re-excision and systemic chemotherapy. That provides reasonable disease control and acceptable toxicity. The dose fractionation and the target's delineation are still mainly to be standardized.
3	Benjamin H. Kann et al. [10]	Postoperative Radiotherapy Patterns of Care and Survival Implications for Medulloblastoma in Young Children.	2016	27491009	Between the ages of three and eight, a worse survival rate is observed when postoperative radiotherapy, as well as the combination of radiotherapy and chemotherapy, is delayed or postponed.
4	Roger J. Packer et al. [11]	Survival and secondary tumours in children with medulloblastoma receiving radiotherapy and adjuvant chemotherapy: results of Children's Oncology Group trial A9961.	2013	23099653	Children with non-diffuse medulloblastoma who have undergone combination therapy (radiotherapy and chemotherapy) have a high survival rate without recurrence. A small percentage may recur after five years from the day of diagnosis, with recurrence occurring in the primary area. Secondary tumours can occur mainly in tumours of the central nervous system.
5	Sindu Vivekanandan et al. [12]	The UK Experience of a Treatment Strategy for Pediatric Metastatic Medulloblastoma Comprising Intensive Induction Chemotherapy, Hyperfractionated Accelerated Radiotherapy and Response Directed High Dose Myeloablative Chemotherapy or Maintenance Chemotherapy (Milan Strategy).	2015	26274622	Careful development of guidelines and better monitoring of toxicity. Strengthen international cooperation to develop the third phase to compare schemes related to radiotherapy and chemotherapy for patients with poor prognoses.
6	Andrè O. von Bueren et al. [13]	Treatment of Children and Adolescents With Metastatic Medulloblastoma and Prognostic Relevance of Clinical and Biologic Parameters.	2016	27863192	The regimen of chemotherapy, hyperfraction scalable radiotherapy, and maintenance chemotherapy showed a correlation between patient status and biological parameters (WNT / MYCC / MYCN status). The treatment regimen appears safe and feasible, resulting in improved survival rates.
7	Magnus Sabel et al. [14]	Relapse patterns and outcome after relapse in standard-risk medulloblastoma: a report from the HIT-SIOP-PNET4 study.	2016	27423645	This study seeks to determine which treatment intervention is best for children with recurrent medulloblastoma. He claims that surgical resection is indicated for localized relapses.
8	Richard L. Bakst et al. [15]	Reirradiation for recurrent medulloblastoma.	2011	21495027	This study's re-irradiation is promising but is performed with great care without overcoming limitations. Re-irradiation was performed mainly on patients who underwent surgery to remove cancer and on fewer who did not.
9	Oya Karadağ et al. [16]	Evaluation of late effects of postoperative radiotherapy in patients with medulloblastoma.	2015	26690598	The radiotherapy for treating medulloblastoma should be appropriate to give a sufficient dose with the best efficiency, as there appears to be late effect toxicity in these long-term surviving patients.
10	Sei Hwan You et al. [17]	Second primary brain tumours following cranial irradiation for pediatric solid brain tumours.	2013	23571774	Patients that underwent re-irradiation showed longer median survival times than the ones who did not. Acute toxicities were not observed. There might be a correlation between chemotherapy during the first treatment and the interval before developing the second brain tumour. The small study group size could not establish a relationship between clinical data and the development of second brain tumours. Radiation dose and volume seem to matter in developing a second brain tumour.
11	Hongzhen Jiang et al. [18]	Intramedullary metastasis in medulloblastoma: a case report and literature review.	2021	33638654	For the management of the disease, it is estimated that surgery, radiotherapy, and chemotherapy play an essential role in managing the disease.
12	Cynthia Wetmore et al. [19]	Reirradiation of recurrent medulloblastoma: does clinical benefit outweigh risk for toxicity?	2014	25080363	Patients who underwent re-irradiation due to recurrent MB disease were shown to contribute to the overall survival of patients. It can also be a treatment option for patients with a small outbreak of residual disease
13	Maura Massimino et al. [20]	Relapse in medulloblastoma: what can be done after abandoning high-dose chemotherapy? A mono-institutional experience.	2013	23595805	MB treatment is always a challenge for radiotherapists, and the experience of the department shows the tailored treatment to each patient depending on his genetic stratification
14	Laetitia Padovani et al. [21]	Reirradiation and concomitant metronomic Temozolomide: an efficient combination for local control in medulloblastoma disease?	2011	22042276	The combined therapeutic regimen of re-radiotherapy and Temozolomide showed good local control of the disease for a long time in individuals with recurrent MB disease.

According to the results presented in Table 1, we found ten retrospective cohort studies [8-13,17,19-21], two retrospective studies [15,16], one clinical study [14], and one case report [18]. All the studies included a combination of radiotherapy, chemotherapy, or surgical therapy to treat medulloblastoma recurrence.

## Discussion

Medulloblastoma is one of the most common cancers in children. Currently, there is no standard treatment strategy for recurrent medulloblastoma, and options vary. A combination of surgical and oncological therapy is usually offered to these patients. The aim of surgical therapy is to achieve a maximal safe resection. The oncological treatment may include radiotherapy with or without chemotherapy or a plan of high-dose chemotherapy followed by autologous stem cell transplantation [22]. Current literature concerning salvage radiotherapy for relapsed medulloblastoma is scarce and based on heterogeneous series that include pediatric and adult patients, applying widely different treatment regimens. Our study reported a rare case of pediatric medulloblastoma and reviewed available literature to evaluate the outcomes of different courses of re-irradiation techniques.

In our patient, recurrence occurred in the right sub-frontal region detected before surgery and in the right temporal pole revealed at MRI examination after surgery. Re-operation of the lesion in the right sub-frontal region, re-irradiation with VMAT-SIB technique, and chemotherapy with temozolomide were performed. We used a hypofractionated regimen of radiotherapy of 50.6Gy given in 22 fractions at the right temporal pole lesion and 39.6 Gy in 22 fractions at the surgical bed. Only mild toxicities were observed (fatigue, alopecia, and nausea). The patient has been free of disease recurrence and progression for two years. The two-year overall survival of patients with recurrent medulloblastoma has been reported to be as low as 25% [23-25]. Based on the treatment plan employed in this case, we can conclude that a combination of approaches and re-irradiation offer good survival outcomes after recurrence for this patient.

Our review of fourteen studies showed that re-irradiation of recurrent medulloblastoma had gained widespread clinical interest due to its improved survival outcomes. The retrospective case series by Tsang et al. reported a median total dose of 58.5Gy (50.5-82.9) and 60.6Gy (51.8-82.9) after re-irradiation of recurrent medulloblastoma at the spinal level and the brain, respectively. Overall median survival was 12.4 months, and the mean time-to-progression was 3.1 months. Spinal re-irradiation was linked with a shorter survival as it was indicated for diffuse disease [8]. Gupta et al. also reported promising results of either a hyperfractionated craniospinal irradiation therapy of 36Gy or re-radiation therapy of 42.5Gy in medulloblastoma patients also undergoing platinum-based chemotherapy. The overall two-year survival was 51%, and progression-free survival was 46% [9]. Similarly, Bakst et al. reported re-irradiation with a median dose of 30Gy (19.8-45Gy) and high-dose chemotherapy after re-resection of recurrent medulloblastoma with overall disease-free survival of 46% at five years after initial recurrence [15]. The median cumulative total dose of radiotherapy to the brain or spine was 84Gy (65-98.4Gy), and intensity-modulated radiation therapy (IMRT) was used in 54% of the cases reported by Bakst et al. [15]. Higher re-irradiation dosage was attempted in the study by Wetmore et al., where eleven children with recurrent medulloblastoma were treated with 36Gy (18-54Gy) of either CSI, spinal or primary-site-only radiotherapy, adding up to a total of 91.9Gy (73.8-109.8Gy). The median overall survival (OS) of these patients was estimated to be 5.39 years (0.3-11.9 years), which is higher than the OS reported in the other studies [19]. Another small retrospective series by Padovani et al. with five children receiving re-irradiation therapy and Temozolomide for recurrent medulloblastoma showed that the combination of these therapies for salvage treatment could offer improved outcomes. The patients were treated with a re-irradiation dose ranging from 20 to 36Gy (1.8Gy per fraction) over five fractions a week, and the five-year event-free survival ranged from 70% to 83% [21].

The results of our study agree with published literature on the efficacy and safety of re-irradiation therapy for children with recurrent medulloblastoma. The risk of neurotoxicity with a median dose of 50.6 Gy (fractional dose: 2.3 Gy/number of fractions: 22) seems to be limited, considering the few side effects our patient presented with. Previous studies suggested that combining re-irradiation and chemotherapy after re-resection of recurrent medulloblastoma may offer the most improved survival outcomes [26]. In particular, a small cohort study has suggested that oral temozolomide and etoposide are two of the most effective chemotherapy agents against recurrent medulloblastoma [27]. Multi-drug anti-angiogenic (anti-VEGF) therapy with a regimen consisting of five drugs has also been found to be beneficial as an adjuvant to re-irradiation for recurrent medulloblastoma [28].

Stratification of patients with molecular subgroup analysis of their medulloblastoma histologic subtype as well as other biologic factors such as specific gene amplifications may help identify patients with a high risk of disease recurrence and progression and, therefore, treatment planning for these particular individuals. For example, von Bueren et al. showed that older children with large cell or anaplastic medulloblastoma (LC/A MB) might have poorer outcomes than children with classic (CMB) or desmoplastic/nodular medulloblastoma (DMB) [13]. In addition, MYC gene amplifications, such as in children with group 3 MYCC/MYCN-amplified medulloblastoma tumors, predispose patients to relapse and poor survival [14]. Future research should systematically report the subgroup analysis of medulloblastoma patients in order to offer patient-centered treatment according to their stratified risk.

The strength of this study is the up-to-date review of literature on the outcomes of re-irradiation for children with recurrent medulloblastoma and the presentation of a case with improved progression-free survival following re-irradiation according to the existing literature. However, the retrospective nature of the studies included in our review, along with their relatively small sample size and lack of molecular stratification of their cohort, introduce selection bias in our findings. Prospective, randomized, multicenter longitudinal studies comparing re-irradiation dose regimens for pediatric recurrent medulloblastoma in a molecularly-stratified subgroup-specific manner are needed to determine the safety and efficacy of re-irradiation and an evidence-based regimen protocol for that.

## Conclusions

Our experience through the case we presented demonstrates that using radiotherapy for a locally-relapsed disease can be a safe 'salvage' treatment offering improved clinical outcomes for children with recurrent medulloblastoma. However, a longer follow-up time period is required to examine long-term survival outcomes. Further research, including third-phase randomized controlled trials, must investigate the appropriate type and dose of re-irradiation therapies for molecularly-stratified patients with recurrent medulloblastoma. The development and universal adoption of guidelines are essential for managing recurrent medulloblastoma with careful monitoring of toxicities.
